# Monitoring of Transplanted Liver Health by Quantification of Organ-Specific Genomic Marker in Circulating DNA from Receptor

**DOI:** 10.1371/journal.pone.0113987

**Published:** 2014-12-09

**Authors:** Hada C. Macher, Gonzalo Suárez-Artacho, Juan M. Guerrero, Miguel A. Gómez-Bravo, Sara Álvarez-Gómez, Carmen Bernal-Bellido, Inmaculada Dominguez-Pascual, Amalia Rubio

**Affiliations:** 1 Dpt. of Clinical Biochemistry, Instituto de Biomedicina de Sevilla (IBiS), Hospital Universitario Virgen del Rocio/CSIC/Universidad de Sevilla, Seville, Spain; 2 Hepatobiliary and Liver Transplantation Unit, Virgen del Rocío University Hospital, Seville, Spain; University of Pisa, Italy

## Abstract

**Background:**

Health assessment of the transplanted organ is very important due to the relationship of long-term survival of organ transplant recipients and health organ maintenance. Nowadays, the measurement of cell-free DNA from grafts in the circulation of transplant recipients has been considered a potential biomarker of organ rejection or transplant associated complications in an attempt to replace or reduce liver biopsy. However, methods developed to date are expensive and extremely time-consuming. Our approach was to measure the SRY gene, as a male organ biomarker, in a setting of sex-mismatched female recipients of male donor organs.

**Methods:**

Cell-free DNA quantization of the SRY gene was performed by real-time quantitative PCR beforehand, at the moment of transplantation during reperfusion (day 0) and during the stay at the intensive care unit. Beta-globin cell-free DNA levels, a general cellular damage marker, were also quantified.

**Results:**

Beta-globin mean values of patients, who accepted the graft without any complications during the first week after surgery, diminished from day 0 until patient stabilization. This decrease was not so evident in patients who suffered some kind of post-transplantation complications. All patients showed an increase in SRY levels at day 0, which decreased during hospitalization. Different complications that did not compromise donated organs showed increased beta-globin levels but no SRY gene levels. However, when a donated organ was damaged the patients exhibited high levels of both genes.

**Conclusion:**

Determination of a SRY gene in a female recipient's serum is a clear and specific biomarker of donated organs and may give us important information about graft health in a short period of time by a non-expensive technique. This approach may permit clinicians to maintain a close follow up of the transplanted patient.

## Introduction

Liver transplantation is a safe and effective treatment for end-stage liver disease. About 6,000 liver transplants are performed in the U.S. each year while another 5000 transplants are included annually in the European Liver Transplant Registry. Prevention and treatment of procedure complications have led us to achieve successful results.

The most frequent complications during the first year are infections and techniques as well as acute cellular rejection that compromise function and graft survival. Most of them are vascular and biliary complications, acute rejection, primary malfunction and dysfunction, as well as the recurrence of HCV [1].

The utility of current liver biomarkers in liver evaluation of transplantation continues to be limited. Thus, they lack specificity when both a lytic (elevated GOT and GPT) or cholestatic (elevated GGT, alkaline phosphatase and Bilirubin) pattern prevails. Differential diagnosis includes vascular or biliary complications, acute rejection, immunosuppressive drug toxicity, preservation injury and infection. Liver biopsy provides diagnostic confirmation of many graft complications although it involves a risk of morbidity (1%) and mortality (from 0.1 to 0.01%) for the patient [2]. Besides, the pathological diagnosis is delayed several hours after sample collection, and, on some occasions, the treatment is initiated in the absence of histological confirmation.

Considerable effort has been made to develop non-invasive techniques that might replace or reduce the need for performing liver biopsies, focusing on monitoring liver functions to detect the onset of graft injury and complications. Thus, several possible biomarkers have been described, either blood (IL, biliary acids, TNF, ICAM-1, hialuronic acid, eosinophilia, B2-microblobulin) or biliary markers (IL-2, IL-6, ICAM-1, cellularity changes). Unfortunately, none of these has provided enough sensitivity and specificity to be reliable biomarkers that permit avoiding histological studies [3], [4]. Currently, studies are focused on directly assessing transplanted organ health. Therefore, instead of monitoring the immune response of an organ recipient, techniques are being developed that inform directly on the health and function of the graft.

Cell free DNA (cfDNA) has been exhaustively studied in the last years as a potential diagnostic, prognostic and monitoring tool in a variety of clinical situations [5]. cfDNA from grafts in the circulation of transplant recipients may be a potential biomarker of organ rejection or transplant associated complications. Thus, instead of monitoring the recipient's immune response we might directly interrogate the health of the donated organ. Recently, Snyder et al reported an increase in specific heart donor cfDNA in the circulation of stable heart transplant recipients during rejection episodes [6]. They developed a sequence based on a technique that involves measuring the signature of dying cells from the organ in the circulating cfDNA in the recipient's plasma. These authors identified a unique genomic DNA signature from the donated organ (compared with the recipient's genome) and they monitored the cfDNA level from the transplanted organ over time to detect possible changes in the donor cfDNA level that correlated with organ health. More recently, the measurement of circulating donor cfDNA by digital PCR expressed by the percentage of plasma circulating cfDNA in relation to that from the recipient has been described [7]. However, to be clinically useful the methods used for the detection of graft cfDNA must not only be specific and sensitive, but they must also be cost-effective and should possibly be performed in a short period of time to be a useful tool for clinicians during the evaluation of transplanted patients. Therefore, some methods described to date are extremely time-consuming and expensive to perform. Some research groups have identified cfDNA in sex-mismatched female recipients of male donor organs, where the chromosome Y can serve as the donor genetic signature. The clearest evidence has come from renal transplantation, where donor-specific chromosome Y has been detected in recipient urine and plasma [8]–[11]. Despite this approach limiting the study to the cases in which a woman receives a transplant of donor male, it is a reliable method that may be performed in routine biochemistry analysis in/after only 3 hours for evaluation of transplanted patient

The aim this study was to evaluate the usefulness of quantifying specific liver cfDNA in the serum of transplanted patients as a non-invasive diagnostic genomic biomarker in the early diagnosis of graft function and complications. The SRY gene, a specific Y-chromosomal gene, has been described as a useful tool for the detection of fetal gender in maternal plasma as well as other chromosome Y genes such as the SRY, DYS14 and DAZ genes [12], [13]. The determination of SRY and DYS14 sequences together is an accurate approach for qualitative diagnosis with sensibilities up to 98%. However the choice of DYS14 gene for quantitative analysis is not appropriated because this gene is present in the genome in tandem with uncertain copy number. Opposite to that one copy of SRY gene is present in the cell genome and may offer a more adequate quantization without interpersonal bias. Besides, SRY sequences have been detected in women's urine and plasma on renal transplantation of sex-mismatched donor–recipients [8]. Thus, in this study we chose the SRY gen as a male organ biomarker in a setting in which the donor was male and the recipient female. We also attempt to associate SRY circulating levels with the clinical evolution of the patients so as to assess its usefulness in the monitoring of post-transplantation liver damage.

## Materials and Methods

### Study Subjects

All consecutive women patients undergoing male liver orthotopic transplantations at the Virgen del Rocio University Hospital in Seville were included in the study. When patients were included in the liver transplant waiting list, they were informed about the study, and they signed a consent form when they were included in the study. Our study included 10 liver transplantation patients who underwent their operations between January 2012 and December 2013. General characteristic of the patients are shown in Table 1. Patients who underwent orthotopic liver transplantation fulfilled the following inclusion criteria: a) be over 18 years old, b) sign the informed consent, c) be a female recipient d) receive a male liver transplantation. One patient (patient 10), although she had suffered a first transplantation from a female donor, was later re-transplanted with a male one and therefore, was also included in the study.

**Table 1:** Characteristics of patients.

Case	Recipient Age	Donor age	Indication of OLT	Recipient BMI	MELD score	Complication
1	57	59	Cryptogenic cirrhosis	26	15	None
2	47	57	HCV cirrhosis	28	25	None
3	66	78	Primary biliary cirrhosis	31	11	None
4	64	37	NASH	29	19	None
5	28	65	Alcoholic cirrhosis	26	20	None
6	54	72	Cryptogenic cirrhosis	22	22	None
7	40	78	HBV cirrhosis + hepatocarcinoma	32	8	Re-transplanted due to hepatic artery thrombosis
8	61	58	Primary biliary cirrhosis	27	20	Cholangitis
9	43	52	Alcoholic cirrhosis	23	19	Re-transplanted due to hepatic vein thrombosis
10	60	64	NASH	33	18	Retrasplantated due to hepatic artery thrombosis

(OLT: orthotopic liver transplantation, BMI: body mass index, HCV: hepatitis c virus, NASH: non-alcoholic steatohepatitis).

During surgery, a blood sample from the central circulation were collected both before and 15 minutes after reperfusion of the new implanted organ. Patients were evaluated clinically and biologically during their Intensive Care Unit (ICU) stay and once they were discharged in doctors' visits. Blood samples were drawn daily during the first three days, and after that samples were taken each two or three days. At this time points a 10 mL blood sample was drawn by peripheral venipuncture to motinor the circulating DNA as well other blood samples for standard biochemical, haematologic and coagulation parameters analyses, and inmunosupressive drug levels determination. In all cases, serum samples were centrifuged within 6 hours after removal and a serum sample was aliquoted and frozen at −80°C for future determination of circulating DNA. During the first week patients also underwent daily abdominal Doppler-ultrasound. After the first week patient evaluation was carried out every 48 hours.

The following post-liver transplantation complications were considered: a) the diagnosis of a vascular complication (portal, hepatic vein or hepatic artery thrombosis) established on the basis of angioCT findings; b) the diagnosis of a biliary complication (stricture, leak, etc) established on the basis of ultrasound, colangioMR and ERCP findings; c) the diagnosis of an acute rejection established on the basis of histopathologic findings in graft biopsy; d) the diagnosis of Graft dysfunction or malfunction established on the basis of biochemical parameters and clinical significance.

### Ethics Statement

Ethical approval for the study protocol was granted by the Medical Research Ethics Board of the Virgen del Rocío University Hospital at Seville. The clinical investigation was conducted according to the principles expressed in the Declaration of Helsinki.

### DNA extraction from serum samples

DNA from 400 µL of serum samples was extracted with the automatized ManNa Pure Compact Instrument (Roche Diagnostics, Bassel, Switzerland) by using Magna Pure Compact Nucleic Acid Isolation Kit I, according to the protocol “Total NA Plasma 100 400 V3 1”. DNA is eluted in a final volume of 50 µL and frozen at −80°C until qPCR.

### Quantification of cfDNA by Real-time quantitative PCR (qPCR)

Serum DNA was measured using a real-time quantitative PCR (qPCR) assay using a Light-Cycler 480 Real-Time PCR instrument (Roche Diagnostics, Bassel, Switzerland). We quantified SRY gene as an specific chromosome Y gene and beta- globin gene as a control of general damage or patient suffering [5], [14]. qPCR analysis was performed by 5′ nuclease assay (hydrolysis probes assay). Two microliters of DNA were amplified in a final volume of 20 µL by using LC480 ProbesMaster Kit (Roche Diagnostics, Bassel, Switzerland) according to the manufacturer instructions. The β-globin hydrolysis probes system consisted of the amplification primers beta-globin Forward (5′-GTG CAC CTG ACT CCT GAG GAG A-3′), beta-globin-Reverse (5′-CCT TGA TAC CAA CCT GCC CAG-3′), and a dual-labeled fluorescent hydrolysis probe betaglobin- (5′-(FAM) TCT GGC CAA GTT TCA ACT CTG CTC GCT -3′ BBQ) and the SRY hydrolysis probe system consisted of the amplification primers SRY Forward (5′-TGGCGATTAAGTCAAATTCGC-3′), SRY Reverse (5′-CCCCCTAGTACCCTGACAATGTATT-3′) and the SRY hydrolysis probe (SRY (FAM) AGCAGTAGAGCAGTCAGGGAGGCAGA -3′ BBQ). qPCR It is performed at 95°C 5″ and 62°C 20″ for 48 cycles. Final size of the amplicon was 102 bp for β-globin and 127 bp for SRY. The standards for the calibration curve based on dilutions of genomic DNA Control Kit (Roche Diagnostics, Bassel, Switzerland).

### Statistical Analysis

Statistical analysis was performed using the Statistical Package for the Social Sciences software package (SPSS 11, Chicago, IL, USA). For comparisons among more than two continuous variables a Kruskall Wallis test followed by the Dunn's Multiple Comparison test for post hoc intra-group analysis were used.

## Results

### Circulating beta-globin levels of patients after liver transplantation

**Figure 1:**
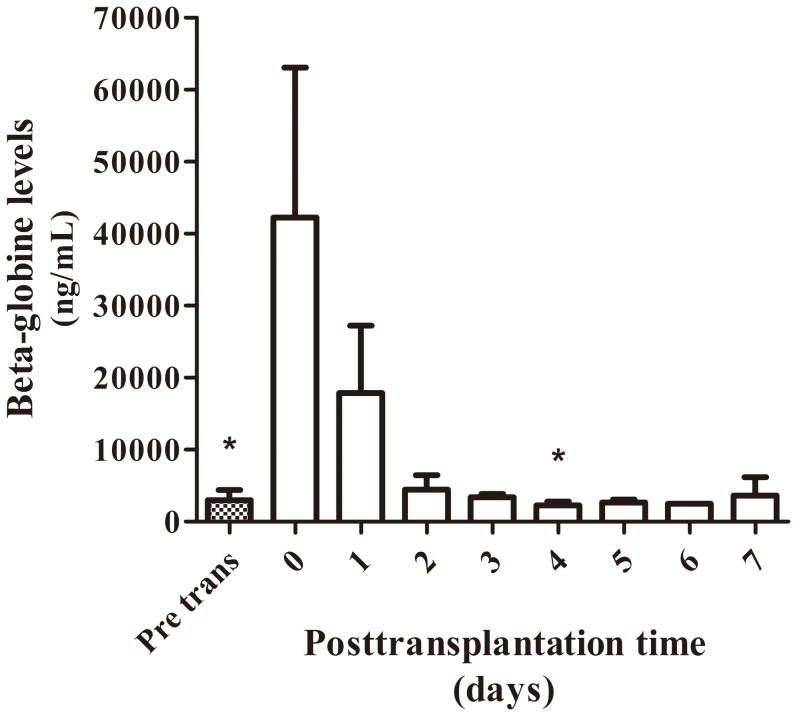
Concentration of serum beta-globin levels in patients without complications. Mean concentration of serum beta-globin circulating levels from patients who accepted transplanted livers without any complications during the first week of stay at ICU (patient 1 to 6, mean +SEM). (*) Kruskall Wallis test, p<0.05; Dunn's Multiple Comparison Test, p<0.05 vs day 0.

**Figure 2:**
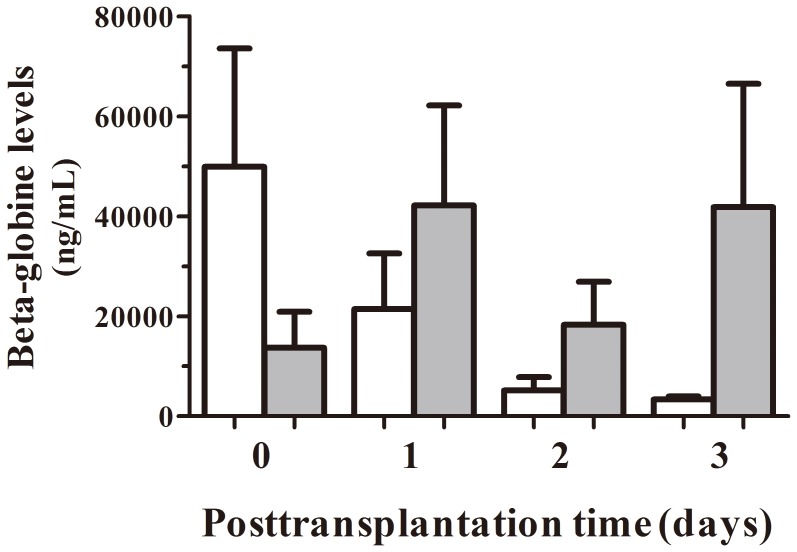
Comparison of serum beta-globin levels in patients with and without complications. Early mean concentration of serum beta-globin circulating levels from patients who accepted transplanted livers without any complications (white bars; n = 6) and patients suffering any kind of post-transplantation complications during their stay at ICU (shadow bars; n = 4).

Six patients accepted the graft without any complications during their stay at ICU. Beta-globin cfDNA was measured as a control of general damage or patient suffering. In Fig. 1 we observed beta-globin mean values during the first week after surgery of these patients. cfDNA levels from samples taken during surgery, at the moment of organ reperfusion, showed a great increase that diminished until patient stabilization. The comparison of the decrease in beta-globin levels between this group of patients and patients who suffered some kind of post-transplantation complications during the first two weeks after transplantation shows a clearly different profile (Fig. 2). Thus, during the first 3 days of follow up, the beta-globin levels in patients with complications were more variable and did not rapidly diminish as did the other group.

### Circulating SRY levels and beta-globin levels of female patients after a male liver transplantation

**Figure 3:**
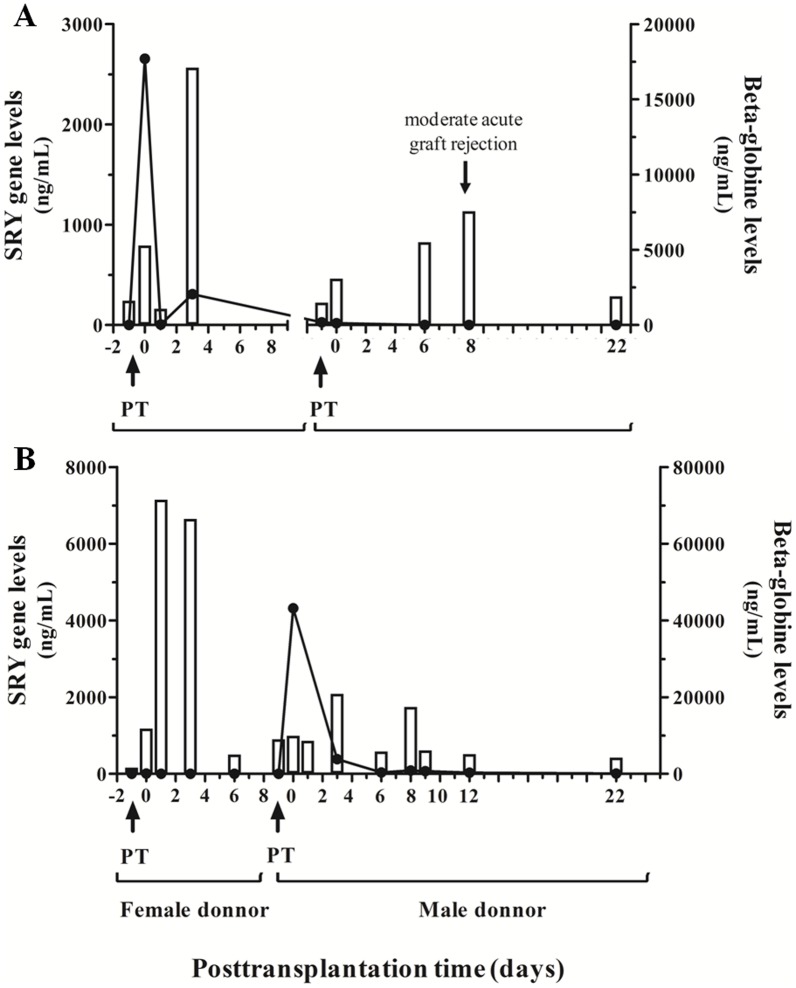
Serum SRY gene and beta-globin gene circulating levels in two re-transplanted female patients. Concentration of serum SRY gene and beta-globin gene circulating levels in two female patients who underwent two consecutive transplantations, one sex-mismatched donor–recipient and the other sex-matched. A) woman who underwent a liver transplantation from a male donor and re-transplanted from a female donor due to hepatic veins thrombosis complication. B) woman who underwent a liver transplantation from a woman donor and re-transplanted from a male donor due to a hepatic artery thrombosis. PT: pre-transplantation sample before organ reperfusion.

SRY gene levels should provide more specific information about the acceptance of the graft than beta-globin levels, a general biomarker of cell damage. We followed up two female patients that suffered re-transplantation due to complications during the first weeks after the first liver transplant (patients 9 and 10). Fig. 3 A shows a woman who underwent a liver transplantation from a male donor and re-transplanted after two months from a female donor due to hepatic veins thrombosis complication at day three after transplantation. This patient showed an early peak of both genes at day 0 that immediately disappeared. On the day of hepatic failure (day 3), the levels of beta-globin greatly increased in this patient due to general clinical damage. Besides, SRY cfDNA levels also increased after the hepatic veins thrombosis as a reflection of the organ failure. After one week, the patient was stabilized and became better and routine liver biomarkers aspartate aminotransferase (GOT), alanine aminotransferase (GPT) and bilirubin levels diminished (Table 2). Due to the liver venoocclusive disease, the patient developed refractarius ascites (diagnosed by liver biopsy) and she was included in waiting list to liver retransplantation. After two months a female liver was transplanted and, as expected, SRY gene amplification was not detected. During the follow up after the second transplant the patient suffered a moderate acute graft rejection (grade II) on day eight after the second transplantation. A lytic hepatocellular lesion was detected, which was attributable to damage from organ reperfusion and revascularization. The patient satisfactorily responded to immunosuppressor therapy and finally accepted the donated organ. As illustrated in fig. 3A and Table 2, an increase in beta-globin levels was observed as early as two days before graft rejection diagnosis. Bilirubin levels were also elevated at this time point. However, GPT values slightly increased on the same day of graft rejection.

**Table 2:** Routine liver biomarkers level and cfDNA values from patient 9.

Day	b-globin (ng/mL)	SRY (ng/mL)	GOT (U/L)	GPT (UI/l)	Bilirubin (mg/dL)
**Pre-trans**	1610	0	49	22	4,6
**0**	5280	2655	3631	1359	1,37
**1**	1080	5,525	1616	1097	1,25
**3^a^**	17100	307	56	582	1,29
**5**	nd	nd	nd	256	2,12
**7**	nd	nd	27	98	2,95
**14**	nd	nd	14	33	1,09
**Pre-trans**	1480	28	nd	10	0,79
**0**	3080	18	nd	302	1,34
**2**	nd	nd	342	186	1,9
**4**	nd	nd	nd	68	6,53
**6**	5500	0	nd	90	13,93
**8^b^**	7570	0	nd	119	12,67
**22**	1910	0	nd	22	4,6

(a) Hepatic veins thrombosis.

(b) Moderate acute graft rejection.

nd: non determined.

The opposite case is observed in fig. 3B, a woman who underwent a liver transplantation from a woman donor and re-transplanted from a male donor due to a hepatic artery thrombosis during surgery. This patient suffering artery thrombosis after first transplantation of a female liver showed an increase in beta-globin gene levels but not in SRY gene ones. High GPT and bilirubin values were observed during this period of time (Table 3). After a second successful transplantation at day 9, SRY amplification peak at day 0 was observed. This patient suffered a biliary peritonitis during re-transplantation showing a more evident increase in beta-globin levels at day 2 of the second transplantation. These data were observed as being parallel with rising values of total bilirubin levels (Table 3). After one week of re-transplantation, an increase in beta-globin levels was also observed probably due to the infection of surgical wound. However, as the liver was not damaged either by the biliary peritonitis nor the surgical infection, SRY gene level did not increase during the complete follow up of the patient.

**Table 3:** Routine liver biomarkers level and cfDNA values of patient 10.

Day	b-globin (ng/mL)	SRY (ng/mL)	GOT (U/L)	GPT (UI/l)	Bilirubin (mg/dL)
**Pre-trans**	1620	0	nd	37	1,44
**0^a^**	11800	0	nd	1431	2,69
**1**	71500	0	51	2042	1,92
**3**	66500	0	438	1032	1,45
**6**	5020	0	49	204	5,5
**9**	8999	0	41	128	4,74
**Pre-trans**	8999	0	41	488	3,76
**0**	9920	4320	nd	493	3,73
**1^b^**	8590	0	1321	417	2,7
**3**	20900	382	113	222	2,92
**6**	5800	39,3	19	71	1,36
**8^c^**	17500	86,9	37	89	1,53
**9**	6090	69,69	24	59	1,05
**12**	5170	28,65	15	36	0,77
**22**	4230	10,28	11	18	0,71

(a) Patient suffered a hepatic arterial thrombosis.

(b) biliary peritonitis.

(c) Infection of surgical wound.

**Figure 4:**
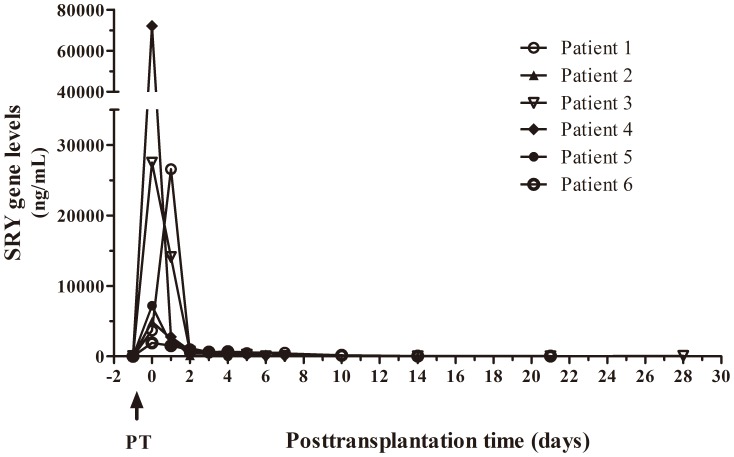
Concentration of serum SRY levels in patients without complications. SRY gene circulating levels of six patients after successful male liver transplantation without any additional complications during one month of follow up.

All patients showed an increase in SRY levels at the moment of transplantation that decreased until patient stabilization. Fig. 4 shows SRY levels in six patients after successful male liver transplantation without any additional complications during recovery at ICU. The early high increase in SRY levels also dropped until basal levels a few days after surgery.

**Figure 5:**
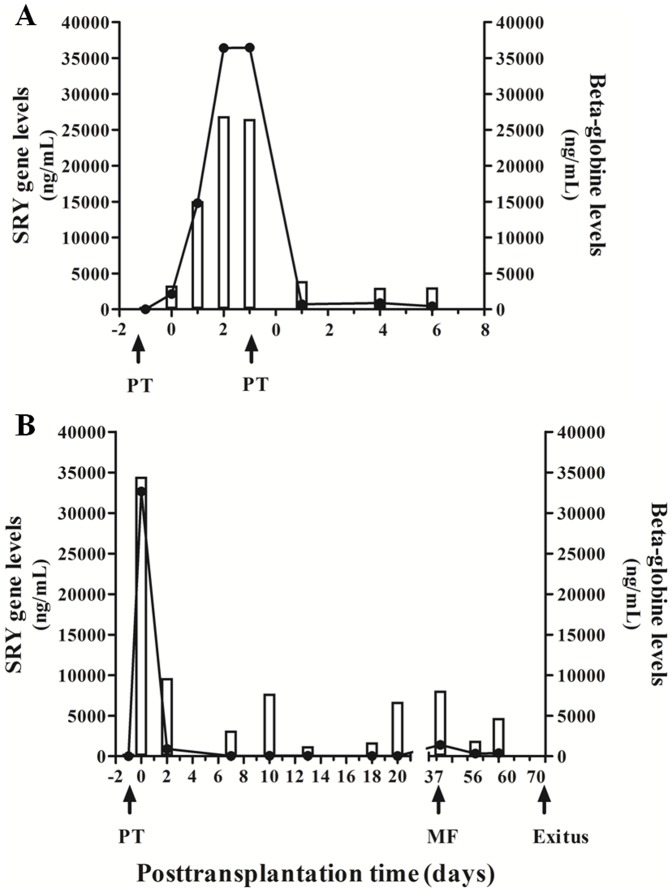
Profile of SRY and beta-globin genes circulating levels of two patients with severe complications after transplantation. A) Patient 7 was transplanted due to a co-infection of HBV and delta virus and after arterial thrombosis was urgently re-transplanted. B) Patient 8 suffered sustained a biliary complication after transplantation that ended in cholestasis. Patient was discharged on day 23 but she was re-admitted to the ICU and suffered a multi-organic failure (MF) on day 37. Patient died on day 70 after transplantation.

Two patients had serious complications after the liver transplantion, patient 7 was urgently re-transplanted 48 h after the first surgery, and patient 8 suffered a sustained choleostasis that finally led to the patient's death. Fig. 5 shows the profile of SRY and beta-globin levels of these two patients with severe complications. Patient 7 suffered cirrhosis due to a co-infection of hepatitis B virus (HBV) and delta virus. Fig. 5A shows high levels of beta-globin gene levels and SRY gene after the first transplantation that decreased after the second. The first transplantation failed due to an arterial thrombosis during surgery and high increases in both SRY and beta-globin gene levels are shown until day two. Routine liver biomarkers GOT and GPT were also highly increased (Table 4). The patient underwent an acute re-transplantation at this moment also from a male donor and circulating cfDNA from both genes diminished. However, after one week of follow up, both biomarkers were a slightly elevated when compared to other patients (Day 6 after re-transplantation: beta-globin levels  = 370 ng/mL and SRY gene levels  = 446 ng/mL) due to complications with the drainage of the biliary conduit. Besides, bilirubin levels also rose over normal values after the second transplantation (Table 4).

**Table 4:** Routine liver biomarkers level and cfDNA values from patient 7.

Day	b-globin (ng/mL)	SRY (ng/mL)	GOT (U/L)	GPT (UI/l)	Bilirubin (mg/dL)
**Pre trans**	353	0	598	396	0,73
**0**	3320	2140	629	470	0,66
**1^a^**	15100	14800	862	857	0,36
**2**	26900	36400	1617	1850	0,38
**Pre trans**	26900	36400	1125	1435	0,38
**4^b^**	3930	711	119	605	2,32
**7**	3000	878	91	401	2,53
**9**	3070	446	59	260	1,1

Aspartate aminotransferase (GOT); alanine aminotransferase (GPT).

(a) Patient suffered an arterial thrombosis.

(b) biliary conduit complication.

Liver transplantation was successful for patient 8, showing low levels of SRY gene during most of the first weeks after surgery (fig. 5B). However, the beta-globin levels continued to be elevated due to a sustained biliary complication that was endoscopically treated and patient 8 clinically improved after two weeks of surgery. The patient was discharged on day 23 after transplantation. During this period, liver biomarkers GOT and GPT, and choleostasis biomarkers (bilirubin levels) were diminishing until she was discharged from ICU (Table 5), although beta-globin levels continues to be elevated. She was re-admitted to the ICU because of sepsis caused by biliary obstruction on day 28 and, finally, a cholestasis was diagnosed that developed a multi-organic failure on day 37. Bilirubin levels were persistently elevated after this point (Table 5). Beta-globin gene levels increased even more at this time point (from 6740 to 8100 ng/mL). Besides, SRY levels that were undetectable during the prolonged stay at ICU were highly increased at the moment of multi-organic failure (1414 ng/mL). The progressive deterioration of the patient at ICU finally led to her death 79 days after transplantation.

**Table 5:** Routine liver biomarkers level and cfDNA values from patient 8.

Day	b-globin (ng/mL)	SRY (ng/mL)	GOT (U/L)	GPT (UI/l)	Bilirubin (mg/dL)
**Pre trans**	320	0	nd	51	10,75
**0**	34500	32650	23	49	12,83
**2**	9640	907,6	nd	649	3,58
**4**	nd	nd	nd	263	2,68
**7**	3170	41,87	19	83	1,73
**10**	7730	61,3	24	58	1,51
**13**	1250	58	42	59	1,34
**18**	1720	35,2	35	64	1,37
**20**	6740	45,7	32	56	1,33
**22^a^**	nd	nd	32	61	1,22
**28^b^**	nd	nd	63	279	12,67
**37^c^**	8010	1414	26	81	14,4
**54**	nd	nd	nd	49	8,01
**56**	1940	335	nd	53	8,28
**59**	4710	374	159	101	15,67
**79^d^**	nd	nd	nd	3	23,86

Aspartate aminotransferase (GOT); alanine aminotransferase (GPT).

(a) The patient was discharged from ICU the next day (day 23 after transplantation).

(b) Patient readmission.

(c) Diagnosis of cholestasis and multi-organic failure.

(d) Patient death.

## Discussion

Nowadays, cfDNA has become a promising tool in diagnosis, prognosis and monitoring of different human pathologies [15]. Cell death has been generally accepted to be an important cause for the release of DNA into the plasma [16]. In this way, some authors have hypothesized that the measurement of donor-derived cfDNA in the plasma of transplant recipients might be used for monitoring graft injury [17]. This hypothesis has subsequently been shown to be correct by different independent workers [6]–[11]. However, the usefulness of this strategy depends on the ability to design molecular assays that allow the detection of donor-specific cfDNA sequences with approaches that provide information to clinicians in a short period of time in order to close monitoring graft health. In this work we used Y-chromosomal markers to evaluate liver transplantation in a setting in which the donor is male and the recipient is female. This same strategy has been previously reported in some patients under renal transplantation [11].

Beta-globin gene cfDNA is a good biomarker of general damage or deterioration of the patient [5], [14]. During transplantation process many possible complications ranking from infection, biliary obstruction to acute rejection, may occur and compromise transplantation success. Although in this work a low number of patients were studied, we observed a diminishing profile of this biomarker when there is no complication after liver transplantation while a more variable pattern of beta-globin cfDNA during the first 72 hours was observed when patients suffered some kind of post-transplantation complications.

After liver transplantation several non-rejection complications can increase the values of commonly used liver function tests that are used as rejection biomarkers. This approach of quantifying cfDNA might be helpful and more specific than routine liver biomarkers. In our opinion, beta-globin gene circulating levels is an unspecific biomarker of cellular damage and does not distinguish between specific graft damage and any other complication during the stay at ICU. The other gene quantified, SRY gene, is shown to be more specific when reflecting liver damage. With the purpose of distinguishing between general and specific damage, we evaluate two patients that underwent re-transplantation, one sex-mismatched donor–recipient and the other sex-matched. One patient underwent a first liver transplantation from a male donor and a second transplantation from a female one (patient 9) while the other patient was transplanted with a male organ and subsequently with a female one (patient 10). Beta-globin increased levels were observed both when the donated organ was damaged and when any other transplantation associated complications occurred. Detectable levels of SRY gene were only observable when patients suffered transplantation from a male donor.

Our results show that SRY is a specific marker of transplanted liver. Thus, when the liver comes from a female donor even if there is a liver damage, with clear increases of beta-globin levels, SRY cfDNA always shows undetectable values. That is the case of patient 9 who suffered a moderate acute rejection of a female organ, showing increased beta-globin levels at day eight but with undetectable levels of SRY gen. Even though the patient had received a male liver in the first transplantation two months before, SRY levels were not detectable at any point due to the rapid clearance of cfDNA [18]. Thus, cfDNA has been generally described as a biomarker of acute processes. An interesting case is patient 8. During the patient follow up the cholangitis suffered during her stay at ICU resulted in a multi-organic failure. At this point the patient exhibited high levels of both genes, with an increase in SRY levels of 1414 ng/µl. Therefore, determination of SRY circulating levels in addition to another non-specific biomarker such as beta-globin levels may allow us to distinguish between specific graft injury and general patient damage.

Even if beta-globin levels may not be the best biomarkers in monitoring liver transplantation, we consider the evidence that low levels of beta-globin circulating gene were always followed by a good patient evolution as interesting. Different complications that do not compromise donated organ showed incrased beta-globin levels but no SRY gene levels, as occurred with patients 8 and 10. Thus, patient 8 shows sustained levels of beta-globin circulating gene during the complete follow up, in concordance with her persistent health deterioration that ended in patient death. However, liver biomarkers and choleostasis biomarkers were diminishing since transplantation and patient temporarily left the hospital, being readmitted due to a general health worsening. Moreover, at the moment of multiorganic failure, although bilirrubine levels were elevated, routine liver biomarkers (GPT and GOT) did not shown any increase Patient 10 suffered a biliary peritonitis during re-transplantation and maintained continuously increased beta-globin cfDNA accompanied of rising values of total bilirubin levels. As a consequence of a surgical wound, an increase in beta-globin levels was detected after one week of re-transplantation. As expected, this lesion did not affect routine liver biomarkers. As either biliary peritonitis or the infection of surgical wound do not compromise donated organ, SRY gene was not detected during the complete 44 days of follow up.

The limitation of this strategy is that it only allows us to work in the subset of transplantation cases with sex-mismatched donor–recipient configuration. However, determination of chromosome Y genes in a female recipient's serum, as SRY gene, turns out to be a clear and specific biomarker of donated organ. Therefore, this specific subset of patients may give us important information about graft health in a short period of time, less than three hours, by a non-expensive technique, with the possibility of it being mechanized. This approach may permit clinicians to maintain a close follow up of the transplanted patient. In a recent publication Snyder et al have reported the determination of graft integrity by DNA sequence methods [6]. The main advantage of this method is that it can be used for all genetically non-identical donor- recipient. However, this method is more expensive and too time consuming to be clinically useful for patient monitoring. In addition, the relative complexity of bioinformatics analysis, and the need of qualified technical workers may be another disadvantage when compared with conventional PCR-based detection strategies.

We address an absolute quantification of organ derived cfDNA with qPCR assay instead of a relative quantification. While the strategies based on sequencing measure the donor-derived cfDNA as a fraction of the total cfDNA in plasma [6], an approach based on PCR can measure an absolute concentration of donor-derived cfDNA in plasma [11], [19]. The measurement of the donor-derived cfDNA as a fraction may be a disadvantage when trying to improve sensitivity as total plasma cfDNA as well as the donor-derived cfDNA will be increased when compared with approaches based only on the detection of the absolute concentration of donor-derived cfDNA [20].

Other authors have measured the percentage of plasma circulating graft DNA in relation to that from the recipient by digital PCR [7]. This technique involves pre-amplification followed by conventional real-time PCR of cfDNA and it attempts to detect heterologous single nucleotide polymorphisms that are used for quantification of graft derived cfDNA as a percentage of total DNA. They reported on identifying both the early rejection and the vascular causes of graft damage at reasonable costs and within a working day. However, the devices that are capable of doing automated digital PCR are not always available in clinical laboratories. Even though this technique is less expensive than sequence based ones, it still cannot be considered cost-effective and suitable for instauration in the routine transplantation monitoring at many hospitals. In the present work, we propose a feasible cost-effective approach (20€/patient determination) that can be carried out in a short period of time (less than 3 hours) that may be suitable for a subset of patients

On the other hand, some authors have suggested that different organs might release different amounts of DNA into the plasma [21]. Therefore, one would predict that the diagnostic threshold concentration for detecting rejection would need to be established individually for different types of organ transplantation. Although we have a low number of patients to establish safe thresholds for liver transplanted patients, our results suggest that amounts of less than 150 ng/mL SRY cell-free circulating concentrations reflect no compromising situation in the organ. Thus, mean value of SRY gene circulating levels of patients without any complications was 133.2±49.6, being 87% of the cases under the mean value.

It has been proposed that increases in organ specific cfDNA during the reperfusion phase after transplantation should be evaluated in order to examine whether these early measurements and the dynamics in the decline of these concentrations, as they may reflect the severity of organ damage [7]. The grade of ischemia/reperfusion damage is still not clearly associated to ultimate long-term graft outcomes. In the present study the number of patients included did not allow us to establish a relationship between the early SRY gene circulating levels during reperfusion and the outcome of liver transplantation. Besides, it might be biased when an intra-surgery sample is collected, because during the surgery some patients receive hemoderived transfusions and may cause inter-patient variation of cfDNA when compared with non-transfused ones.

CfDNA has become an important tool for early diagnosis in many pathologies by replacing proteomic approaches for genomic ones. Thus, an exciting approach is also the ability to use direct sequencing of plasma cfDNA to identify the mutations in cell-free tumor DNA, effectively transforming a blood sample into a “liquid biopsy.”[22]. In this context, monitoring graft health by specific organ cfDNA has turned out to be a non invasive and advantageous approach. Further research should be performed in order to find an organ genomic signature longer than an SNP to get robust quantification in a short period of time and with easily available techniques as occurs with SRY gene detection. This approach may be helpful to detect and possibly prevent early liver rejection and in the future, it may have the potential to complement or possibly even replace other approaches used for post-transplant monitoring. Thus, personalized monitoring by measurement of specific organ cfDNA may be an effective tool to accomplish personalized immune-suppression as well as rejection monitoring for earlier intervention and prevention.

## References

[1] Agopian VG, Petrowsky H, Kaldas FM, Zarrinpar A, Farmer DG, et al (2013) The evolutions of liver transplantations during 3 decades, analysis of 5347 consecutive liver transplants at a single center. Ann Surg 258:409–21. 24022434 10.1097/SLA.0b013e3182a15db4

[2] Grant A, Neuberger J (1999) Guidelines on the use of liver biopsy in clinical practice. Gut 45 (suppl IV): IV 1–IV 11. 10.1136/gut.45.2008.iv1 PMC1766696 10485854

[3] Millán O, Rafael-Valdivia L, Torrademé E, López A, Fortuna V, et al (2013) Intracellular IFN-γ and IL-2 expression monitoring as surrogate markers of the risk of acute rejection and personal drug response in de novo liver transplant recipients. Cytokine 61(2):556–64. 23265966 10.1016/j.cyto.2012.10.026

[4] Vivarelli M, Smith HM, Naoumov NV, Williams R (1995) Quantitative assessment of serum beta-2-microglobulin in liver transplant recipients and relationship to liver graft rejection. Eur J Gastroenterol Hepatol 7(12):1215–9. 8789315 10.1097/00042737-199512000-00016

[5] Macher H, Egea-Guerrero JJ, Revuelto-Rey J, Gordillo-Escobar E, Enamorado-Enamorado J, et al (2012) Role of early cell-free DNA levels decrease as a predictive marker of fatal outcome after severe traumatic brain injury. Clin Chim Acta 24(414):12–7. 10.1016/j.cca.2012.08.001 22902808

[6] Snyder TM, Khush KK, Valantine HA, Quake SR (2011) Universal noninvasive detection of solid organ transplant rejection. Proc Natl Acad Sci U S A 108:6229–34. 21444804 10.1073/pnas.1013924108 PMC3076856

[7] Beck J, Bierau S, Balzer S, Andag R, Kanzow P, et al (2013) Digital droplet PCR for rapid quantification of donor DNA in the circulation of transplant recipients as a potential universal biomarker of graft injury. Clin Chem 59:1732–1741. 24061615 10.1373/clinchem.2013.210328

[8] Zhang J, Tong KL, Li PK, Chan AY, Yeung CK, et al (1999) Presence of donor- and recipient-derived DNA in cell-free urine samples of renal transplantation recipients: Urinary DNA chimerism. Clin Chem 45:1741–1746. 10508119

[9] Zhang Z, Ohkohchi N, Okazaki H, Guo Y (2003) Use of PCR and PCR-SSP for detection of urinary donor-origin DNA in renal transplant recipients with acute rejection. Chin Med J (Engl) 116:191–194. 12775227

[10] Zhong XY, Hahn D, Troeger C, Klemm A, Stein G, et al (2001) Cell-free DNA in urine: a marker for kidney graft rejection, but not for prenatal diagnosis? Ann N Y Acad Sci 945:250–257. 11708487

[11] García Moreira V, Prieto García B, Baltar Martín JM, Ortega Suárez F, Alvarez FV (2009) Cell-free DNA as a noninvasive acute rejection marker in renal transplantation. Clin Chem 55:1958–1966. 19729469 10.1373/clinchem.2009.129072

[12] Mortarino Garagiola I, Lotta LA, Siboni SM, Semprini AE, Peyvandi F, et al (2011) Non-invasive tool for foetal sex determination in early gestational age. Haemophilia 17:952–6. 21492325 10.1111/j.1365-2516.2011.02537.x

[13] Macher H, Noguerol P, Medrano-Campillo P, Garrido-Márquez MR, Rubio-Calvo A, et al (2012) Standardization non-invasive fetal RHD and SRY determination into clinical routine using a new multiplex RT-PCR assay for fetal cell-free DNA in pregnant women plasma: results in clinical benefits and cost saving. Clin Chim Acta 18 413:490–4. 10.1016/j.cca.2011.11.004 22133782

[14] Rainer TH, Wong LKS, Lam W, Yuen E, Lam NYL, et al (2003) Prognostic use of circulating plasma nucleic acids concentrations in patients with acute stroke. Clin Chem 49:562–569. 12651807 10.1373/49.4.562

[15] Yu SC, Chan KC, Zheng YW, Jiang P, Liao GJ, et al. (2014) Size-based molecular diagnostics using plasma DNA for noninvasive prenatal testing Proc Natl Acad Sci USA May 19. pii: 201406103. [Epub ahead of print]. 10.1073/pnas.1406103111 PMC4060699 24843150

[16] Tong YK, Lo YM (2006) Diagnostic developments involving cell-free (circulating) nucleic acids. Clin Chim Acta 363(1–2):187–96. 16126188 10.1016/j.cccn.2005.05.048

[17] Lo YMD, Tein MSC, Pang CCP, Yeung CK, Tong KL, et al (1998) Presence of donor-specific DNA in plasma of kidney and liver-transplant recipients. Lancet 351:1329–30. 9643800 10.1016/s0140-6736(05)79055-3

[18] Yu SC, Lee SW, Jiang P, Leung TY, Chan KC, et al (2013) High-Resolution Profiling of Fetal DNA Clearance from Maternal Plasma by Massively Parallel Sequencing. Clin Chem 59:1228–37. 23603797 10.1373/clinchem.2013.203679

[19] Gadi VK, Nelson JL, Boespflug ND, Guthrie KA, Kuhr CS (2006) Soluble donor DNA concentrations in recipient serum correlate with pancreas-kidney rejection. Clin Chem 52:379–82. 16397013 10.1373/clinchem.2005.058974

[20] Lo YM (2011) Transplantation monitoring by plasma DNA sequencing. Clin Chem Jul 57(7):941–2 doi:10.1373/clinchem.2011.166686 10.1373/clinchem.2011.166686 21566070

[21] Lui YYN, Woo KS, Wang AYM, Yeung CK, Li PKT, et al (2003) Origin of plasma cell-free DNA after solid organ transplantation. Clin Chem 49:495–6. 12600963 10.1373/49.3.495

[22] Forshew T, Murtaza M, Parkinson C, Gale D, Tsui DW, et al (2012) Noninvasive identification and monitoring of cancer mutations by targeted deep sequencing of plasma DNA. Sci Transl Med 4:136ra68.. 10.1126/scitranslmed.3003726 22649089

